# Semi-supervised bidirectional alignment for Remote Sensing cross-domain scene classification

**DOI:** 10.1016/j.isprsjprs.2022.11.013

**Published:** 2023-01

**Authors:** Wei Huang, Yilei Shi, Zhitong Xiong, Qi Wang, Xiao Xiang Zhu

**Affiliations:** a Chair of Data Science in Earth Observation, Technical University of Munich, Munich, 80333, Germany; b Chair of Remote Sensing Technology, Technical University of Munich, Munich, 80333, Germany; c School of Artificial Intelligence, Optics and Electronics (iOPEN), Northwestern Polytechnical University, Xi’an, 710072, China

**Keywords:** Remote sensing, Semi-supervised domain adaptation, Cross-domain classification, Bidirectional sample-class alignment

## Abstract

Remote sensing (RS) image scene classification has obtained increasing attention for its broad application prospects. Conventional fully-supervised approaches usually require a large amount of manually-labeled data. As more and more RS images becoming available, how to make full use of these unlabeled data is becoming an urgent topic. Semi-supervised learning, which uses a few labeled data to guide the self-training of numerous unlabeled data, is an intuitive strategy. However, it is hard to apply it to cross-dataset (i.e., cross-domain) scene classification due to the significant domain shift among different datasets. To this end, semi-supervised domain adaptation (SSDA), which can reduce the domain shift and further transfer knowledge from a fully-labeled RS scene dataset (source domain) to a limited-labeled RS scene dataset (target domain), would be a feasible solution. In this paper, we propose an SSDA method termed bidirectional sample-class alignment (BSCA) for RS cross-domain scene classification. BSCA consists of two alignment strategies, unsupervised alignment (UA) and supervised alignment (SA), both of which can contribute to decreasing domain shift. UA concentrates on reducing the distance of maximum mean discrepancy across domains, with no demand for class labels. In contrast, SA aims to achieve the distribution alignment both from source samples to the associate target class centers and from target samples to the associate source class centers, with awareness of their classes. To validate the effectiveness of the proposed method, extensive ablation, comparison, and visualization experiments are conducted on an RS-SSDA benchmark built upon four widely-used RS scene classification datasets. Experimental results indicate that in comparison with some state-of-the-art methods, our BSCA achieves the superior cross-domain classification performance with compact feature representation and low-entropy classification boundary. Our code will be available at https://github.com/hw2hwei/BSCA.

## 1 Introduction

Remote sensing (RS) image scene classification (Wang et al., 2020, Cheng et al., 2017, Bai et al., 2022, Ma et al., 2021a, Xiong et al., 2022) has attracted increasing attention for its broad real-world applications, such as land use (Ma et al., 2017) and urban planing (Rottensteiner et al., 2012, Qiu et al., 2019). However, most state-of-the-art methods of RS image scene classification are based on fully-supervised deep learning models, which depend heavily on numerous manually-labeled data. In contrast, more and more unlabeled RS images have accumulated and are accessible to researchers. In this situation, the question of how to utilize unlabeled RS images effectively has become an urgent problem to be solved.

Several semi-supervised learning (SSL) works (Han et al., 2018, Miao et al., 2022) on RS image scene classification have been attempted in order to reduce the heavy dependence on annotations by self-training on the unlabeled data. However, they cannot directly take advantage of the large-scale labeled data of other existing datasets for the cross-dataset (cross-domain) shifts. Concretely, there are many factors resulting in domain shifts across RS scene classification datasets, such as different times, various scales, different capturing equipment, and unfixed shooting angles. In practice, the mixture of these factors further complicates domain shifts. On the other hand, some unsupervised domain adaptation (UDA) methods have been explored for RS cross-domain scene classification (Ma et al., 2021b, Zheng et al., 2022b, Yu et al., 2022, Ahmed et al., 2021, Wurm et al., 2019), aiming to utilize the existing labeled RS image dataset (source domain) to help the classification of the unlabeled RS image dataset (target domain) within a shared class space. However, the generalization of UDA is limited due to the lack of labeled target data, which is essential for reducing the domain shift as a bridge between the source domain and the target domain. From this point of view, as a combination of SSL and UDA, semi-supervised domain adaptation (SSDA) would be a feasible solution, as it can transfer knowledge from an annotation-rich source domain to a marginally-labeled target domain under the guidance of a few labeled target data. Still, similar to UDA, SSDA suffers from domain shift.

Feature alignment is an empirically effective way to decrease domain shift, and can therefore boost the classification performance of the target domain. From the perspective of feature levels, current feature alignment of SSDA can be roughly divided into two types, sample alignment (Motiian et al., 2017) and domain alignment (Kim and Kim, 2020). Sample alignment aims to align cross-domain intra-class instances, while domain alignment tries to align the global feature distribution of the two domains. As their intermediate strategy, class alignment is more robust than sample alignment for its low sensitivity to noisy samples and more fine-grained than domain alignment because of its focalization operations. For an accurate and stable class alignment, it is necessary to calculate the class center from multiple samples at the training stage. Unfortunately, the number of the samples within the same class is quite limited in each mini-batch, which obstructs the accurate and robust calculation of class-wise centers as well as the further class alignment.

To achieve the flexible and robust class-level feature alignment without a high computing burden, we devise a novel holistic SSDA framework termed as bidirectional sample-class alignment (BSCA) for RS cross-domain scene classification, which consists of two feature alignment modules:

(1) an unsupervised alignment (UA) module is introduced to achieve the global distribution alignment between the feature extracted from labeled samples (containing source samples and labeled target samples) and the feature extracted from unlabeled target samples, with no demand for their class information. It is achieved by decreasing the distance of maximum mean discrepancy (MMD) (Gretton et al., 2012a, Pan et al., 2010) not only between the source domain and the unlabeled target domain but also between the labeled target domain and unlabeled target domain;

(2) an original supervised alignment (SA) module is proposed to achieve feature alignment from samples to their corresponding cross-domain class centers. Here, class centers are calculated by memory bank mechanism that can individually store the feature extracted from source samples and target samples. In SA, there are two separated memory banks, source memory bank and target memory bank, corresponding to two kinds of class centers. Taking the feature extracted from a source sample as an example, it plays two roles simultaneously at each training iteration: (a) it is used to update the source memory bank of the same class; and (b) it is aligned to the target class center of the same class, which is calculated from the target memory bank. Similarly, the feature extracted from a target sample is used to update the target memory bank, and is aligned to the corresponding source class center. Therefore, our SA can achieve the feature alignment both from source samples to target centers and from target samples to source centers, i.e., bidirectional alignment. Besides, benefiting from memory bank, abundant class-aware feature of historical samples can be stored and used for real-time sample-class feature alignment with a small amount of computing resources. In other words, the SA of BSCA can calculate the robust class centers from abundant class-aware feature beyond the limitation of mini-batch and support the class-wise feature alignment in an online manner.

To objectively evaluate the effectiveness of the proposed BSCA, we build an RS-SSDA benchmark with the common classes of four widely-used RS image scene classification datasets, and then compare our method with some state-of-the-art SSL, UDA, and SSDA methods on this benchmark. Experimental results indicate that our BSCA outperforms the comparison methods and achieves the best mean classification performance of a total of 12 adaptation scenarios in the benchmark. Overall, our contributions can be summarized as follows:

• We propose a bidirectional sample-class alignment (BSCA) method for RS-SSDA, consisting of two modules of supervised alignment (SA) and unsupervised alignment (UA). SA aims to achieve the bidirectional feature alignment both from source samples to target class centers and from labeled/pseudo-labeled target samples to source class centers. By contrast, UA focuses on the global alignment among the features extracted from labeled samples and unlabeled samples without a demand for their class information.
• To verify the effectiveness of the proposed method, we collect an RS-SSDA benchmark dataset with 7 common classes of 4 widely-used RS image scene classification datasets, and implement extensive experiments based on it.
• Compared with some state-of-the-art SSL/UDA/SSDA methods, our proposed BSCA obtains the best mean classification results on the above RS-SSDA benchmark, demonstrating the effectiveness of our method.

## 2 Related work

### 2.1 Semi-supervised domain adaptation

SSDA is a recently rising research field, which can be seen as a combination of SSL and UDA. To our best knowledge, there are roughly three kinds of SSDA methods: adversarial training based SSDA (Tzeng et al., 2017, Jiang et al., 2020), entropy optimization based SSDA (Grandvalet et al., 2005, Saito et al., 2019, Li and Hospedales, 2020), and feature alignment based SSDA (Motiian et al., 2017, Kim and Kim, 2020, Li et al., 2021). Specifically, Tzeng et al. (2017) proposed a Siamese architecture for addressing the SSDA problem and the generalization of deep models. This architecture learns a discriminative embedding subspace by introducing a classification and contrastive semantic alignment (CCSA) loss to handle the feature of labeled source and target samples; however, it ignores the usage of unlabeled target samples. Saito et al. (2019) highlighted this field by a novel minimax entropy (MME) approach that adversarially optimizes a SSDA model; MME can alternately maximize the conditional entropy of unlabeled target data for the feature classifier and minimize it for the feature encoder. Jiang et al. (2020) devised a general bidirectional adversarial training (BiAT) to guide adversarial examples across the domain gap. Li and Hospedales (2020) proposed an online shortest-path meta-learning framework that is computationally tractable and effective in practice for both multi-source unsupervised domain adaptation (MSDA) and SSDA. Aiming to align features by reducing of the intra-domain discrepancy, Kim and Kim (2020) presented an SSDA framework mainly consisting of three schemes of attraction, perturbation, and exploration. Li et al. (2021) devised am approach termed cross-domain adaptive clustering (CDAC) to achieve both inter-domain and intra-domain adaptation, via introducing an adversarial adaptive clustering loss to group features of unlabeled target data into clusters and then implementing cluster-wise feature alignment across domains.

### 2.2 Domain adaptation of RS scene classification

In recent years, an increasing number of works have focused on the domain adaptation (DA) of RS image scene classification (Song et al., 2019, Lu et al., 2020, Zheng et al., 2021, Zhang et al., 2020, Zhu et al., 2021, Zheng et al., 2022a, Lasloum et al., 2021). In Song et al. (2019), a new subspace alignment layer added into CNN models was proposed for the DA of RS image scene classification to align the source domain and the target domain in the feature subspace; as a result, it can optimize CNN models to adapt to the classification of the target domain. In Zhang et al. (2020), a correlation subspace dynamic distribution alignment method was proposed for RS image scene classification, consisting of subspace correlation maximization (SCM) that tries to avoid mapping source domain data into irrelevant subspace, and dynamic statistical distribution alignment (DSDA) that aims to reduce the cross-domain distribution discrepancy. An attention-based multiscale residual adaptation network (AMRAN) was proposed for cross-scene classification tasks (Zhu et al., 2021). In AMRAN, both marginal and conditional distributions were taken into consideration, and the multiscale attention mechanism was used to extract robust features and complete information. In Zheng et al. (2022a), the single-source multiple-target domain adaptation task was explored for RS applications and a new algorithm named two-stage adaptation network (TSAN) was presented, which: (1) utilizes the adversarial learning approach to confuse the classifier between the source domain and the whole mixed multi-target domain, and (2) adopts self-supervised learning to divide the mixed-multiple-target domain with its pseudo domain labels in order to learn intrinsic features of multiple target domains. In Lasloum et al. (2021), the MME algorithm (Saito et al., 2019) mentioned in Section 2.1 was applied to multi-source SSDA for the purpose of RS image scene classification.

Our work mainly concentrates on RS image scene classification in the single-source single-target SSDA setting, with the intent to learn the transferable knowledge from a single-source domain to a single-target domain, in order to obtain the domain-invariant feature representation that would also be helpful for multi-source/multi-target research.

### 2.3 Memory bank

The memory bank module has been broadly applied because it can store extra information outside the neural network and can be used as a dictionary for reference. For example, a memory-augmented temporal bidirectional learning network, which can learn to write the most evident information into an external memory module, was proposed for human action recognition in Yuan et al. (2019). A bidirectional prototype unit was presented to encode the normal dynamics as prototypes for real-time frame construction in Lv et al. (2021). From the perspective of contrastive learning, a bidirectional dictionary was built based on a memory bank with a moving-averaged queue encoder in momentum contrast (MoCo) for unsupervised visual representation learning (He et al., 2020). In Alonso et al. (2021), the memory bank mechanism was combined with contrastive learning to enforce a segmentation network to obtain pixel-level feature representations that are similar to cross-domain intra-class samples for the semi-supervised semantic segmentation; however, it only updates with the feature vectors from labeled data, ignoring the utilization of unlabeled data.

## 3 Methodology

**Fig. 1:**
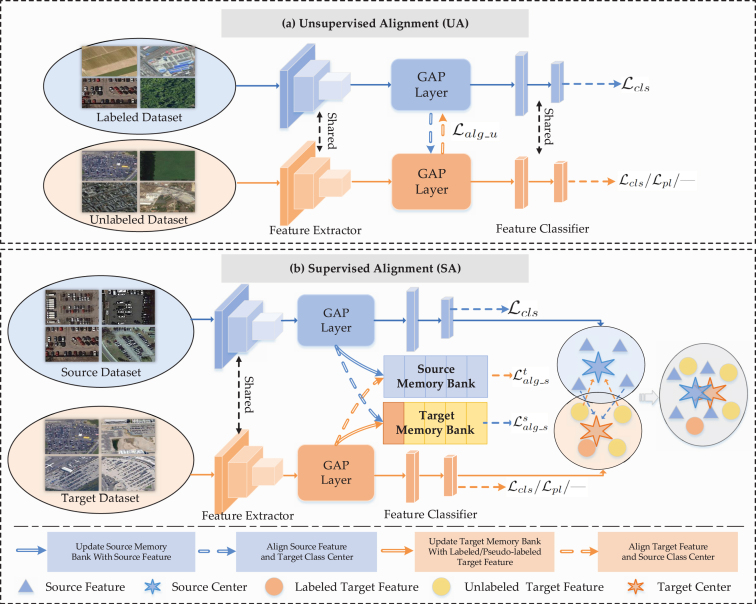
The workflow of the proposed BSCA for RS-SSDA, consisting of two parts: (a) UA that aligns the cross-domain class-agnostic features without their class information; and (b) SA that aligns the cross-domain class-aware features with a demand for their class labels/pseudo-labels.

This section begins by introducing some notations of the universal SSDA, then presents the shared network architecture, and finally describes the proposed BSCA in detail with the summary of its training procedure. The workflow of the BSCA based SSDA architecture is shown in Fig. 1.

### 3.1 Notations

In the setting of SSDA, there are three subdomains: a fully-labeled source domain S, a limited-labeled target domain $T_{l}$, and an unlabeled target domain $T_{u}$. Their sample sets are denoted as $D_{s}={\left\{\left(x^{s},y^{s}\right)\right\}}_{i=1}^{N_{s}}$, $D_{l}={\left\{\left(x^{l},y^{l}\right)\right\}}_{i=1}^{N_{l}}$, and $D_{u}={\left\{\left(x^{u}\right)\right\}}_{i=1}^{N_{u}}$, respectively, where x, y, and N are an image, its associated label, and the sample number of its domain, respectively. Here $N_{l}$ is much smaller than $N_{u}$. Source and target labels, $y^{s}$ and $y^{l}$, have the same class space {1, …, *K*}, where *K* is the number of classes.

### 3.2 Shared network architecture

Following some existing SSDA literature (Chen et al., 2019, Saito et al., 2019), a CNN model is used for RS cross-domain scene classification in this paper. It consists of three components: a CNN based feature extractor E, a global average pooling (GAP) layer G, and a feature classifier C. All of these are shared between the source domain and the target domain.

First, a feature map $m\in R^{H\times W\times C}$, where *H*, *W*, and *C* are the spatial height, spatial width, and feature dimension, respectively, is extracted from an image x by the feature extractor E, which is denoted as

(1) m=E(x).

Then a global feature vector $f\in R^{C}$ is calculated from m via the GAP layer G (Lin et al., 2013) as

(2) $$f=G\left(m\right)=\frac{1}{HW}\overset{H}{\underset{i=1}{\sum }}\overset{W}{\underset{j=1}{\sum }}m_{ij}.$$

Finally f is fed into the feature classifier C to obtain a class-wise probabilistic prediction vector $p\in R^{K}$, which is denoted as

(3) $$\left\{\begin{array}{c} f=\frac{f}{\left\|f\right\|},\\ p=\sigma \left(C\left(f\right)/T\right), \end{array}\right)$$

where the first equation refers to the $l_{2}$ normalization (Ranjan et al., 2017). The term σ is the Softmax function, which can normalize the sum of all the elements of the prediction vector to 1; this means the element value of p can be seen as the probability of the corresponding class. T is a hyperparameter of temperature.

Following the common practice in the SSDA literature (Saito et al., 2019, Li et al., 2021), all the labeled samples from both the source domain $D_{s}$ and the labeled target domain $D_{l}$ are used to train the network via a classification loss (the standard cross-entropy loss) as

(4) $$L_{cls}=-\underset{\left\{x,y\right\}\in D_{s}\cup D_{l}}{\sum }ylogp_{y}.$$

### 3.3 Bidirectional sample-class alignment

BSCA is composed of UA and SA, which aims to achieve the unsupervised and supervised feature alignment as shown in Fig. 1.

#### 3.3.1 Unsupervised alignment

As shown in the “unsupervised alignment” part of Fig. 1, UA mainly concentrates on unsupervised inter-domain alignment between the features extracted from labeled samples and the features extracted from unlabeled samples. The distance of MMD (Gretton et al., 2012a, Pan et al., 2010) in reproducing kernel Hilbert space (RKBS) (Borgwardt et al., 2006) is used to evaluate the mean-value similarity of these two distributions as

(5) $$L_{alg\text{\_}u}=d_{MMD}^{2}={\left\|\frac{1}{N_{s}+N_{l}}\overset{N_{s}+N_{l}}{\underset{i=1}{\sum }}\phi \left(f_{i}^{sl}\right)-\frac{1}{N_{u}}\overset{N_{u}}{\underset{i=1}{\sum }}\phi \left(f_{i}^{u}\right)\right\|}_{H}^{2},$$

where $f^{sl}$ represents the global feature vector extracted by Eqs. (1)–(2) from the images of the source and labeled target domains, and $f^{u}$ represents the global feature vector extracted from the images of the unlabeled target domain. The function ϕ is Gaussian radial basis function (RBF) kernels that can project $f^{sl}$ and $f^{u}$ to the RKBS. To improve the capacity of feature representation, the multiple kernel variant of MMD, i.e., MK-MMD (Gretton et al., 2012b, Long et al., 2015), is used for the distance measurement.

Here it is worth mentioning that instead of the only alignment between the source data and the target data, UA focuses on aligning the feature distribution between all the labeled domains (containing the source domain and the labeled target domain) and unlabeled target domain. The reason for it is that labeled target data can obtain robust and discriminative feature representation by supervised training, and they can work with fully-labeled source data to provide more comprehensive feature distribution at the domain level as a more robust anchor for the features extracted from unlabeled data.

#### 3.3.2 Supervised alignment

As shown in Fig. 1(b), SA aims to realize a feature alignment from samples to cross-domain class centers via the memory bank mechanism.

In SA, there is a source memory bank and a target memory bank, denoted as $M^{s}$ and $M^{t}\in R^{K\times N\times C}$, respectively, to store the features extracted from source samples and target samples. Here K is the class number of the dataset, C is the channel dimension of the feature, and N is the item number of each class in $M^{s}$ and $M^{t}$. The target memory bank $M^{t}$ consists of two sub-components: a labeled target memory bank $M^{l}\in R^{K\times N_{u}\times C}$ and an unlabeled target memory bank $M^{u}\in R^{K\times N_{u}\times C}$, where $N_{l}+N_{u}=N$. These memory banks are initialized at zero.

**Fig. 2:**
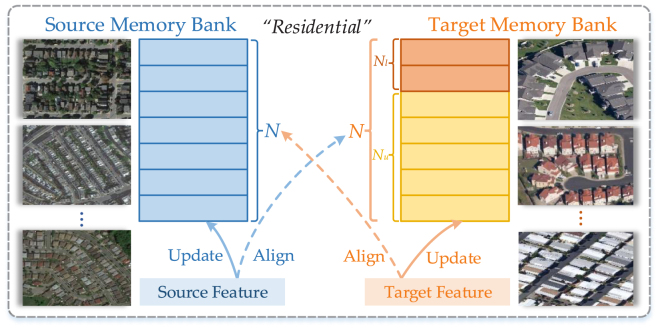
The illustration of SA for RS cross-domain scene classification, with the class “residential” as an example. (For interpretation of the references to color in this figure legend, the reader is referred to the web version of this article.)

The pipeline of the memory bank based SA is depicted in Fig. 2. In the figure, **blue** rectangles represent the features extracted from source images, **orange** rectangles represent the features extracted from labeled target images, and **yellow** rectangles represent the features extracted from high-confidence unlabeled target images. Based on the memory bank, SA combines the advantages of both sample alignment and class alignment, where samples allow flexible optimization and class centers provide stable and robust anchors. Concretely, robust class centers can be calculated from multiple features stored in memory banks, without a demand for the computation-consuming feature extraction process; meanwhile, features extracted from samples of a mini-batch can be aligned to their associated cross-domain class centers in real time. As a result, such sample-class alignment takes up little extra computing resources yet achieves both flexible and robust feature alignment during the training phase.

The detailed process of SA can be divided into the following three steps:

(a) **Generating feature-label pairs**. First, it is necessary to generate the feature-label pair [f, y] at each iteration. Using Eqs. (1)–(2), the images $x^{s}$, $x^{l}$, and $x^{u}$ are input to the combination of E and G, and then the corresponding global feature vectors $f^{s}$, $f^{l}$, and $f^{u}$ are output. Of those three vectors, $f^{s}$ and $f^{l}$ can be directly combined with their associated labels $y^{s}$ and $y^{l}$ to form the feature-label pairs [$f^{s}$, $y^{s}$] and [$f^{l}$, $y^{l}$]. For $f^{u}$, which has no associated label, its maximum-activated class is used as its pseudo-label ${\overset{ˆ}{y}}^{u}$, as

(6) $${\overset{ˆ}{y}}^{u}=\underset{i=\left\{1,\ldots ,K\right\}}{\mathrm{argmax}}p_{i}^{u},$$

where $p^{u}$ is calculated from $f^{u}$ via Eq. (3). The terms $f^{u}$, $p^{u}$, and ${\overset{ˆ}{y}}^{u}$ form the pseudo feature-label pair [$f^{u}$, $p^{u}$, ${\overset{ˆ}{y}}^{u}$].

(b) **Updating memory banks with feature-label pairs**. Then it is important to design a reasonable updating strategy to update the memory banks, which provide anchors for sample-class alignment and therefore determine the performance of SSDA. $M^{s}$ and $M^{l}$ are updated by the corresponding features $f^{s}$ and $f^{l}$; to decrease the interference of noise labels as much as possible, $M^{u}$ is updated by the high-confidence $f^{u}$, whose pseudo-class probability is higher than a threshold τ. The updating strategy is shown in Algorithm 1. Among it, M[y,1: N−1] represents the items from 1 to N−1 of the class y in the corresponding memory bank M. Following the online learning framework of BSCA, memory banks are updated accordingly every iteration to: (1) produce real-time class centers instead of lagging ones, which are consistent with the features extracted from images in real time; and (2) generate flexible items of $M^{u}$, which can avoid the occupation of wrongly pseudo-labeled target samples because they will be replaced iteratively. Another benefit of memory bank is that it can make our BSCA independent of the sample number of classes. Technically, our BSCA only requests at least one labeled source sample and one labeled target sample per class for the calculation of their associate class center.

**Figure:**
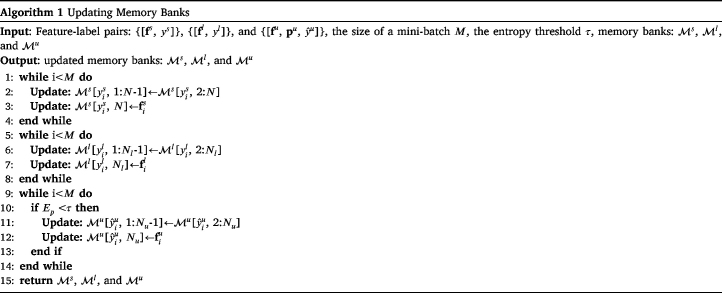

On the other hand, the pseudo-labels of high-confidence unlabeled samples can also be used to train the network to improve the weight of the unlabeled target domain, as

(7) $$\left\{\begin{array}{c} E_{p}=-\overset{K}{\underset{i=1}{\sum }}p_{i}^{u}logp_{i}^{u},\\ L_{pl}=-\underset{x^{u}\in D_{u}}{\sum }1\left\{E_{p}<\tau \right\}\cdot {\overset{ˆ}{y}}^{u}logp_{{\overset{ˆ}{y}}^{u}}^{u}, \end{array}\right)$$

where $1\left\{E_{p}<\tau \right\}$ is the indicator function whose value is 1 when the entropy sum value of $E_{p}$ is smaller than an entropy threshold τ, and is 0 otherwise.

(c) **Making the cross-domain sample-class alignment**. Finally, we make the cross-domain alignment between feature and class centers both from the source domain to the target domain and from the target domain to the source domain. Before alignment, it is necessary to calculate the source and target class centers, denoted as $c^{s},c^{t}\in R^{K\times C}$. The kth class center is the average of all the items of its class in the associated memory bank, which are formulated as

(8) $$\left\{\begin{array}{c} c_{k}^{s}=\frac{1}{N}\overset{N}{\underset{i=1}{\sum }}M_{ki}^{s},\\ c_{k}^{t}=\frac{1}{N}\left(\overset{N_{l}}{\underset{i=1}{\sum }}M_{ki}^{l}+\overset{N_{u}}{\underset{i=1}{\sum }}M_{ki}^{u}\right). \end{array}\right)$$

Here it is worth mentioning that at the beginning of training, there are not enough feature to update the memory banks, which means some items remain in the initial zero state. To prevent their interference, these zero items are not calculated in the class centers during this stage.

The source and target class centers are used as anchors for the alignment of the features $f^{s}$, $f^{l}$, and $f^{u}$. The whole supervised alignment loss $L_{alg\text{\_}s}$ is composed of two directions of the source feature alignment loss $L_{alg\text{\_}s}^{s}$ and the target feature alignment loss $L_{alg\text{\_}s}^{t}$, which are calculated as

(9) $$\left\{\begin{array}{c} L_{alg\text{\_}s}^{s}=\frac{1}{M}\overset{M}{\underset{i=1}{\sum }}\left(f_{i}^{s}-c_{y_{i}^{s}}^{t}\right),\\ L_{alg\text{\_}s}^{t}=\frac{1}{M}\left[\overset{M^{l}}{\underset{i=1}{\sum }}\left(f_{i}^{l}-c_{y_{i}^{l}}^{t}\right)+\overset{M^{u}}{\underset{i=1}{\sum }}\left(f_{i}^{u}-c_{{\overset{ˆ}{y}}_{i}^{u}}^{s}\right)\right],\;\;{\overset{ˆ}{y}}_{i}^{u}>\tau ,\\ L_{alg\text{\_}s}=L_{alg\text{\_}s}^{s}+L_{alg\text{\_}s}^{t}. \end{array}\right)$$

Here the feature-label pairs [$f^{s}$, $y^{s}$], [$f^{l}$, $y^{l}$], and [$f^{u}$, ${\overset{ˆ}{y}}^{u}$] are provided by step (1). Following Li et al. (2021), when $f^{u}$ is used for the alignment, it is replaced by the counterpart extracted from the augmented part of the same target image by the RandAugment technique (Cubuk et al., 2020). Within a mini-batch, $M^{u}$ is the number of the unlabeled feature $f^{u}$ whose pseudo-label ${\overset{ˆ}{y}}^{u}$ is greater than τ, and $f^{u}$ is the closest to $c_{{\overset{ˆ}{y}}_{i}^{u}}^{s}$ using mean square error (MSE) as the measurement. In BSCA, class centers only serve as the anchors for the feature, without the gradient back-propagation to the corresponding raw images. As a result, such feature-class alignment takes up little computing resources during the training phase.

#### 3.3.3 Overall loss and training procedure of BSCA

The overall loss of the BSCA-based SSDA model is the combination of supervised classification loss $L_{cls}$, pseudo-label classification loss $L_{pl}$, unsupervised feature alignment loss $L_{alg\text{\_}u}$, and supervised cross-domain feature-class alignment loss $L_{alg\text{\_}s}$, formulated as

(10) $$L=L_{cls}+L_{pl}+\alpha L_{alg\text{\_}u}+\beta L_{alg\text{\_}s},$$

where α and β are the weights of $L_{alg\text{\_}u}$ and $L_{alg\text{\_}s}$.

For a clear understanding of the workflow of our method, an iteration of the training procedure of BSCA is summarized below. In each iteration, three mini-batches of images, $x^{s}$, $x^{l}$, and $x^{u}$, are randomly sampled from $D_{s}$, $D_{l}$, and $D_{u}$, respectively, and are sent into the shared feature extractor and feature classifier. Accordingly, three mini-batches of global features ($f^{s}$, $f^{l}$, and $f^{u}$) and class-wise predictions ($p^{s}$, $p^{l}$, and $p^{u}$) are obtained. Among them, $p^{s}$ and $p^{l}$ are used for the calculation of the cross-entropy loss $L_{cls}$; the high-confidence parts of $p^{u}$ are used for the calculation of pseudo-label classification loss $L_{pl}$; $f^{u}$ and the concatenation of $f^{s}$ and $f^{l}$ are used for the calculation of unsupervised alignment loss $L_{alg\text{\_}u}$; $f^{s}$ and the concatenation of $f^{l}$ and $f^{u}$ are used for the calculation of supervised alignment loss $L_{alg\text{\_}s}$. Then, the overall loss is obtained from the above losses by Eq. (10), and is used for gradient back-propagation and model optimization. Finally, $f^{s}$ are used for updating the source memory bank, $f^{l}$ are used for updating the labeled target memory bank, and the high-confidence parts of $f^{u}$ are used for updating the unlabeled target memory bank.

**Fig. 3:**
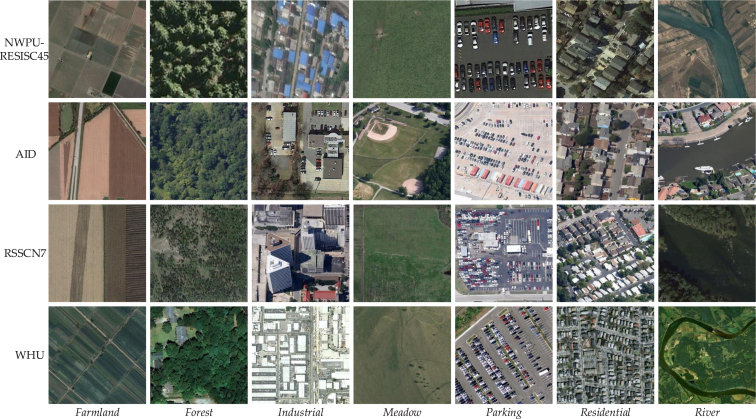
Some examples of the 7 common classes of four RS scene datasets. It could be observed that there are some domain shifts among different datasets, such as various scales and angles.

## 4 Experiments

In this section, some experimental settings are introduced, including benchmark datasets and implementation details. Then two kinds of hyper-parameter tuning experiments are conducted to explore the boundary of the proposed BSCA. Following it, the experiments are conducted on the proposed method in comparison with some state-of-the-art SSL/UDA/SSDA methods. Next, the ablation study is performed to explore the individual effect of each component of BSCA. Finally, visualizations of the feature distributions of BSCA and other methods are provided for intuitive comparison.

### 4.1 Experimental settings

**Benchmark datasets**. To comprehensively evaluate the proposed method, we collect and build an RS-SSDA benchmark with 7 shared classes from four widely-used RS image scene classification datasets: RSSCN7 (Zou et al., 2015), NWPU-RESISC45, AID, and WHU-RS19. Each dataset can serve as both the source domain and the target domain, and therefore there are 12 adaptation scenarios in total. For each dataset, all the images are with their class labels when serving as the source domain, while only random $N_{l}$ images of each class are with labels (labeled target domain) and all the rest images are unlabeled (unlabeled target domain). All the unlabeled target data are used for model training in an unsupervised manner. Among them, 6 samples per class are used as the validation set, and all the rest data are used as the test set. Their released years, capturing resolutions, original image sizes, class names, and sample numbers are given in Table 1, and some examples are shown in Fig. 3. In the following sections, NWPU-RESISC45, AID, RSSCN7, and WHU-RS19 are abbreviated as N, A, R, and W, respectively.

**Implementation details**. We employ the experiments based on three widely-used advanced CNN backbones, VGG16 (Simonyan and Zisserman, 2014), ResNet34 (He et al., 2016), and EfficientNet_B0 (Tan and Le, 2019), all of which are pretrained on ImageNet (Deng et al., 2009). Their feature maps are embedded into the corresponding global feature vectors by GAP layer as Eq. (2) for the follow-up SSDA operation, and their last fully-connected layers are replaced by the feature classifier C described in Eq. (3) with the temperature parameter T set to 0.05, which is consistent with the existing SSDA works (Saito et al., 2019, Kim and Kim, 2020, Li et al., 2021). The item number of source and target memory banks is 32; that of the labeled target memory bank is 2 $\times \;N_{l}$ and thus that of the unlabeled target memory bank is the rest (32-2 $\times \;N_{l}$). In Eqs. (7), (9), the entropy threshold τ is set to 0.5 for all four datasets as the target domain in the ablation, comparison, and visualization experiments. In Eq. (10), the unsupervised alignment loss weight α and supervised alignment loss β are set to 10 and 0.025, respectively. Besides the above specific hyper-parameters of our BSCA, for fair comparison, all the common training settings are kept the same as other SSDA methods used in this paper. Concretely, Stochastic Gradient Descent (SGD) with momentum of 0.9 is used as the optimizer to train the models. Learning rate is initialized at 0.01 and decreases with a weight decay of 0.005, with the mini-batch M size set to 24. Models for all the methods are trained for 2000 iterations in all 12 adaptation scenarios, and they are validated by the validation set every 50 iterations. During the training stage, the ones with best validation performance are saved, and they are tested by the test set after training.

**Table 1:** Characteristics of RS image scene classification sets.

	NWPU-RESISC45	AID	RSSCN7	WHU-RS19
Years	2017	2016	2015	2010

Resolution(m)	0.2–30	0.5–8	–	0.5

Image size	256 × 256	600 × 600	400 × 400	600 × 600

Class 1: *Farmland*	700	370	400	50
Class 2: *Forest*	700	250	400	53
Class 3: *Industrial*	700	390	400	53
Class 4: *Meadow*	700	280	400	61
Class 5: *Parking*	700	390	400	50
Class 6: *Residiential*	700	410	400	54
Class 7: *River*	700	410	400	56

The experiments are implemented based on PyTorch 1.9.1^1^ (Paszke et al., 2017) on two GeForce RTX 2080Ti GPUs.

### 4.2 Ablation study

The whole objective function of the proposed BSCA has four components of supervision classification loss $L_{cls}$ for the labeled data, pseudo-label classification loss $L_{pl}$ for high-confident unlabeled data, unsupervised alignment loss $L_{alg\text{\_}u}$ among unsupervised cross-domain features, and the supervised sample-class alignment loss $L_{alg\text{\_}s}$ among the class-aware/pseudo-class-aware features. To verify their respective effect on the knowledge transfer, this subsection implements an ablation study of BSCA on all 12 adaptation scenarios with ResNet34 as the CNN backbone. Experimental results are provided in Table 2.

**Table 2:** Classification accuracy (%) of ablation study under the 3-shot setting with **ResNet34** as the CNN backbone.

$L_{cls}$	$L_{pl}$	$L_{alg\text{\_}s}$	$L_{alg\text{\_}u}$	A→N	R→N	W→N	N→A	R→A	W→A	N→R	A→R	W→R	N→W	A→W	R→W	*Mean*
✓				90.7	77.4	84.3	93.3	86.0	94.2	75.9	72.5	72.9	89.9	98.3	92.5	*85.7*
✓	✓			94.6	86.5	94.2	95.9	94.7	97.2	78.9	79.0	81.6	97.6	97.9	93.4	*91.0 (＋5.3)*
✓	✓	✓		91.9	**89.8**	93.2	96.7	96.0	96.7	80.5	79.9	82.3	**97.9**	99.0	96.9	*91.7* (+*6.0*)
✓	✓		✓	96.6	89.0	94.5	96.8	96.8	**97.6**	81.6	81.9	**84.1**	**97.9**	99.0	96.2	*92.7 (＋7.0)*
✓	✓	✓	✓	**96.8**	89.7	**94.7**	**97.2**	**97.2**	97.5	**84.1**	**82.6**	84.0	**97.9**	**99.7**	**98.3**	***93.3 (＋7.6)***

**Fig. 4:**
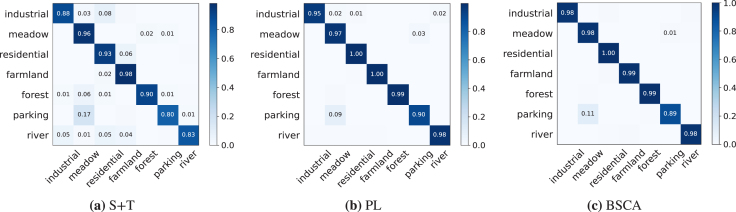
The confusion matrices of the methods of S+T, PL, and the proposed BSCA in the N→A adaptation scenario, with ResNet34 as the CNN backbone.

When only using $L_{cls}$, the mean classification accuracy of 85.7% over all the 12 adaptation scenarios is obtained. After embedding $L_{pl}$, the mean accuracy dramatically increases to 91.7% due to the involution of unlabeled target data, which can reduce the entropy of the classification boundary, and decrease the over-impact of the source domain. In addition, both of $L_{alg\text{\_}s}$ and $L_{alg\text{\_}u}$ contribute to further performance improvement based on $L_{cls}+L_{pl}$ to different extent. It is found that only adding $L_{alg\text{\_}s}$ can obtain a gain of 0.7% over the 91.0% of $L_{cls}+L_{pl}$, probably limited by the incorrectly pseudo-labeled target data. Fortunately, after being combined with $L_{alg\text{\_}u}$, the individual improvement of $L_{alg\text{\_}s}$ further climbs by 0.6%, and therefore the final BSCA obtains the best result of 93.3%, which has an advantage of 7.6% over the baseline of $L_{cls}$ as well as 2.3% over $L_{cls}+L_{pl}$, without adding extra model parameters. The ablation results demonstrate the effectiveness of each component of the proposed BSCA.

Pseudo-label training inevitably leads to noisy labels of $D_{u}$ while improving the classification performance. However, the ablation results reveal that during the feature alignment stage, class centers of the target domain are not negatively affected by these noise pseudo-labels in BSCA. Fortunately, there are five factors that can relieve this problem: (1) source domain and target domain shares the same class space, and therefore the model trained on the source domain can also be used for the target domain to some extent; (2) only high-confidence unlabeled target samples can be used for pseudo-label training, which can largely decrease the impact of wrongly pseudo-labeled target samples; (3) features extracted from labeled target samples occupy a constant number of items in the target memory banks, which provide some pure samples, especially at the early stage; (4) numerous items in the target memory banks can dilute the interference of a small number of noise features by the average operation. Therefore, the update process for the target memory bank is robust enough for the follow-up alignment; and (5) the UA module of BSCA achieves class-irrelevant domain-level alignment between the source domain and the unlabeled target domain, and thus can make the distribution of the latter more stable and less affected by the noise pseudo-labels.

The confusion matrices of S+T ($L_{cls}$), PL ($L_{cls}\;+\;L_{pl}$), and the complete BSCA are shown in Fig. 4. It can be seen that the more components are added, the clearer confusion matrices are obtained, especially for the class of “residential”, which further verifies the effectiveness of our algorithm design.

### 4.3 Hyper-parameter tuning experiments

To further explore the performance boundary of our BSCA, two kinds of hyper-parameter tuning experiments, entropy threshold tuning and shot number (number of labeled samples per class) tuning, are conducted in this subsection.

**Fig. 5:**
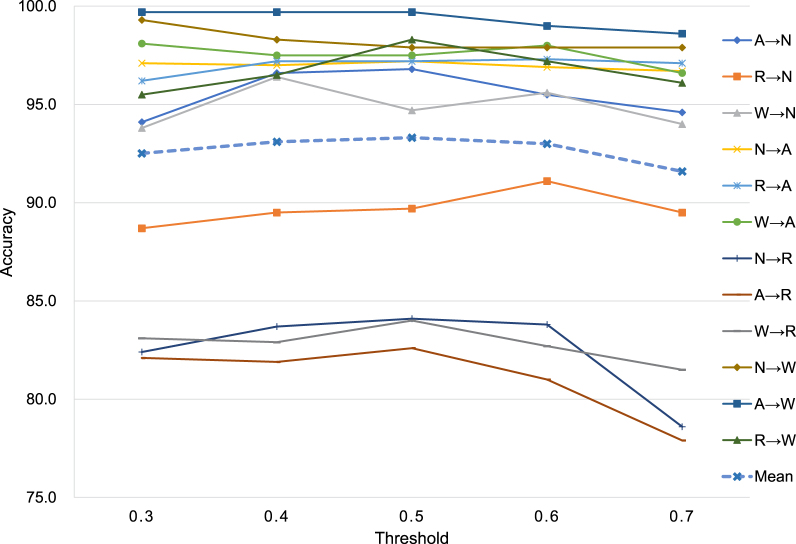
Classification accuracy with different entropy thresholds of the proposed BSCA based on ResNet34.

The experimental results of entropy threshold tuning are reported in Table 3 and plotted in Fig. 5. 0.3 is low and therefore it allows a relatively small number of pseudo-labeled target samples for training, resulting in the under-fitting of the model; by contrast, 0.7 is so high that too many unlabeled data are pseudo-labeled and self-trained, leading to the over-fitting of wrongly pseudo-labeled data. Overall, it could be observed that 0.5 achieves the best mean classification performance from the tendency of Fig. 5. In the following experiments, entropy threshold is set to 0.5 by default.

**Fig. 6:**
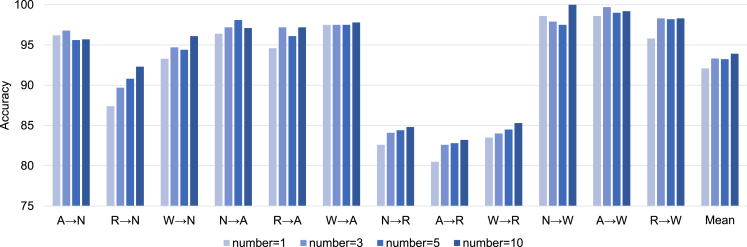
Classification accuracy with different numbers of labeled target samples per class.

On the other hand, the experimental results of shot number tuning is provided in Table 4 and Fig. 6. In general, classification accuracy increases with the growth of shot number especially when the basic performance (1-shot) is under 95%. When shot number increases from 3 to 10, mean accuracy increases by 0.7%, showing limited performance gain. From the perspective of real application, we focus on the experiments under the 3-shot setting, which also follows the practice of some classical SSDA work (Saito et al., 2019, Li et al., 2021).

**Table 3:**
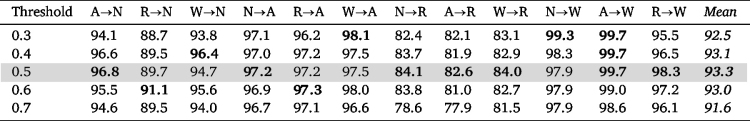
Classification accuracy (%) of threshold tuning experiments under the 3-shot setting with **ResNet34** as the CNN backbone.

**Table 4:**
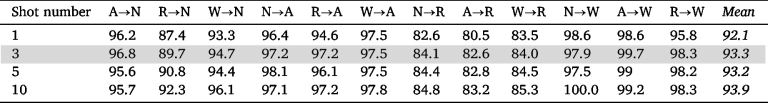
Classification accuracy (%) of different numbers of labeled samples per class with **ResNet34** as the CNN backbone.

### 4.4 Comparison experiments

To fairly and comprehensively evaluate the effectiveness of our BSCA on RS cross-domain scene classification, based on 12 kinds of adaptation scenarios of the proposed RS-SSDA benchmark, we conduct comparison experiments between our method and some state-of-the-art UDA, SSL, and SSDA algorithms on three popular CNN backbones.

#### 4.4.1 Baselines

To verify the advantages of SSDA to SSL/UDA in RS cross-domain scene classification, our BSCA is compared with: (1) **FixMatch** (Xiong et al., 2021, Sohn et al., 2020), one classical SSL method, which was applied to semi-supervised RS scene classification and achieved one of the state-of-the-art results in this field; and (2) **ECB-FAM** (Ma et al., 2021b), one of the latest UDA algorithms for RS cross-domain scene classification. For more comprehensive evaluation, our BSCA is further compared with some SSDA methods reproduced in this study, including: (3) **S+T**, which is the basic method only using source data and labeled target data for training; (4) **ADDA** (Wang et al., 2018, Tzeng et al., 2017), which is the first work on RS-SSDA to our knowledge. It adversarially trains the source data and target data, based on two individual extractors and a shared classifier; (5) **RevGrad** (Lasloum et al., 2021, Lu et al., 2019), which was commonly applied to RS cross-domain scene classification, developed from the method of DANN (Ganin et al., 2016, Saito et al., 2019). It can adversarially decrease the discrimination of the feature classifier on source and target feature via a gradient reverse layer; (6) **SSDAN** (Lasloum et al., 2021), which is the current state-of-the-art approach for RS-SSDA, derived from the method of MME (Saito et al., 2019). It alternatively maximizes the conditional entropy of unlabeled target data for the feature classifier and minimizes it for the feature extractor; (7) **ENT** (Grandvalet et al., 2005, Saito et al., 2019), which is a classical semi-supervised learning method (Grandvalet et al., 2005) widely applied in the universal SSDA field. It aims to decrease the entropy of classification boundary for more confident classification; and (8) **CDAC** (Li et al., 2021), which is one of the state-of-the-art SSDA methods in computer vision, and is applied to RS-SSDA for comparison in this paper. It focuses on both inter-domain and intra-domain adaptation via grouping features of unlabeled target data into clusters across domains.

Here we implement all the SSDA methods based on the official codes (S+T & ENT & SSDAN,^2^ ADDA,^3^ RevGrad,^4^ and CDAC^5^). The robustness of these methods on CNN models are evaluated on three widely-used CNN backbones: (1) **VGG16** (Simonyan and Zisserman, 2014), which is a classical deep CNN architecture for complex visual representation; (2) **ResNet34** (He et al., 2016), which utilizes the skip residual connection to avoid the problem of vanishing gradients; and (3) **EfficientNet_B0** (Tan and Le, 2019), which is designed by the neural architecture search technique, efficiently reducing model parameters.

**Table 5:** Classification accuracy (%) of comparison experiments on the 12 adaptation scenarios from the RS-SSDA datasets under the 3-shot setting with **VGG16** as the CNN backbone. The best results are in **bold**.

Type	Method	A→N	R→N	W→N	N→A	R→A	W→A	N→R	A→R	W→R	N→W	A→W	R→W	*Mean*
SSL	FixMatch (Xiong et al., 2021)	77.9			96.3			67.5			93.4			*83.8*

UDA	ECB-FAM (Ma et al., 2021b)	90.5	74.6	80.6	93.9	79.5	92.3	69.7	71.0	68.2	94.1	97.7	87.6	*83.3*

SSDA	S+T	92.6	83.6	88.0	94.1	88.9	93.8	72.9	72.4	76.9	96.9	98.3	92.4	*87.6*
	ADDA (Wang et al., 2018)	81.8	69.2	73.9	90.5	82.4	77.9	61.8	64.8	66.7	86.1	89.6	80.9	*77.1*
	RevGrad (Lasloum et al., 2021)	88.2	81.7	90.0	93.9	88.9	89.8	73.8	71.9	77.5	96.9	98.3	93.4	*87.0*
	SSDAN (Lasloum et al., 2021)	94.6	84.7	89.5	**96.8**	94.2	95.5	**79.6**	80.6	81.8	98.3	**99.3**	96.9	*91.0*
	ENT (Grandvalet et al., 2005)	86.5	72.0	86.9	92.2	86.0	91.7	60.4	68.0	73.0	80.2	98.6	89.9	*82.1*
	CDAC (Li et al., 2021)	85.6	74.6	71.7	89.9	81.8	79.5	65.0	70.2	69.8	81.2	89.2	73.6	*77.7*

**Our BSCA**	**96.5**	**94.2**	**95.9**	96.5	**94.5**	**96.5**	78.0	**82.8**	**84.7**	**98.6**	99.0	**99.3**	***93.0***

**Table 6:** Classification accuracy (%) of comparison experiments on the 12 adaptation scenarios from the RS-SSDA datasets under the 3-shot setting with **ResNet34** as the CNN backbone. The best results are in **bold**.

Type	Method	A→N	R→N	W→N	N→A	R→A	W→A	N→R	A→R	W→R	N→W	A→W	R→W	Mean
SSL	FixMatch (Xiong et al., 2021)	81.3			95.0			63.5			97.6			*84.4*

UDA	ECB-FAM (Ma et al., 2021b)	88.5	74.5	83.2	92.5	77.5	92.8	66.9	68.9	65.4	91.7	97.6	79.9	81.6

SSDA	S+T	89.9	76.4	84.4	91.7	84.5	91.6	71.4	68.4	69.4	92.0	97.6	91.0	*84.0*
	ADDA (Wang et al., 2018)	87.3	78.6	83.1	77.2	76.5	90.7	64.7	72.4	65.0	89.9	94.8	85.8	*80.5*
	RevGrad (Lasloum et al., 2021)	90.2	80.2	86.9	93.5	86.8	91.7	76.7	73.6	74.1	95.5	98.3	88.5	*86.3*
	SSDAN (Lasloum et al., 2021)	95.5	86.6	93.5	96.9	95.0	97.3	80.2	80.2	83.6	97.3	99.3	96.2	*91.8*
	ENT (Grandvalet et al., 2005)	79.5	67.7	77.2	91.2	91.2	87.5	66.6	60.5	62.6	89.9	91.0	83.7	*85.4*
	CDAC (Li et al., 2021)	85.4	80.6	75.2	83.8	90.0	88.5	65.8	65.5	72.7	86.1	80.6	92.0	*79.8*

**Our BSCA**	**96.8**	**89.7**	**94.7**	**97.2**	**97.2**	**97.5**	**84.1**	**82.6**	**84.0**	**97.9**	**99.7**	**98.3**	***93.3***

**Table 7:** Classification accuracy (%) of comparison experiments on the 12 adaptation scenarios from the RS-SSDA datasets under the 3-shot setting with **EfficientNet_B0** as the CNN backbone. The best results are in **bold**.

Type	Method	A→N	R→N	W→N	N→A	R→A	W→A	N→R	A→R	W→R	N→W	A→W	R→W	*Mean*
SSL	FixMatch (Xiong et al., 2021)	72.2			93.2			63.0			96.9			*81.3*

UDA	ECB-FAM (Ma et al., 2021b)	91.7	76.8	83.0	91.8	80.5	90.8	68.6	69.5	62.4	92.4	98.6	84.7	*82.6*

SSDA	S+T	91.1	81.5	86.0	92.6	84.2	91.4	71.4	71.9	66.2	93.1	97.6	90.4	*84.8*
	ADDA (Wang et al., 2018)	90.2	77.7	84.3	89.1	79.8	90.8	69.8	69.9	69.3	94.1	96.6	83.3	*82.9*
	RevGrad (Lasloum et al., 2021)	90.9	78.4	84.5	92.7	84.4	91.5	72.8	69.6	72.0	94.1	**99.0**	89.2	*84.9*
	SSDAN (Lasloum et al., 2021)	90.6	79.6	83.7	90.8	86.0	89.6	73.1	73.8	72.0	96.5	**99.0**	87.2	*85.2*
	ENT (Grandvalet et al., 2005)	88.3	78.2	84.8	91.5	83.2	90.5	70.7	68.2	71.9	96.5	97.9	91.0	*84.4*
	CDAC (Li et al., 2021)	87.0	84.7	86.0	88.5	78.6	84.8	68.5	66.3	68.5	88.9	92.7	81.2	*81.3*

**Our BSCA**	**96.1**	**88.3**	**93.8**	**96.5**	**95.8**	**96.4**	**77.0**	**78.9**	**83.0**	**98.3**	98.6	**98.6**	***91.8***

#### 4.4.2 Comparison with state-of-the-art methods

Comparison results of VGG16, ResNet34, and EfficientNet_B0 are reported in Tables 5, 6, and 7, respectively. Overall, the proposed BSCA achieves the best performances with respect to mean classification accuracy of the 12 adaptation scenarios of the RS-SSDA benchmark dataset. BSCA obtains mean accuracies of 93.0%, 93.3%, and 91.8% on VGG16, ResNet34, and EfficientNet_B0, respectively, with the performance advantages of 2.0%, 1.5%, and 6.6% over the sub-optimal method of SSDAN.

**Fig. 7:**
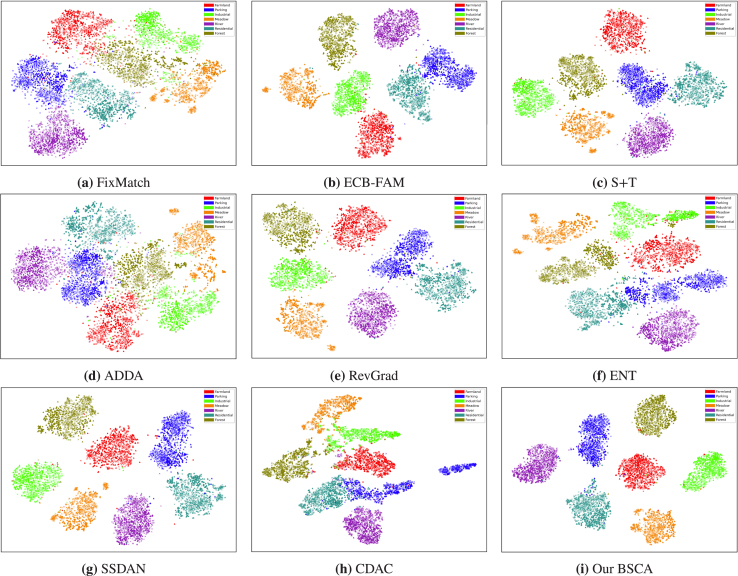
The t-SNE visualizations of the feature distribution of the methods of the proposed BSCA and other comparison methods for the N→A adaptation scenario, with ResNet34 as the CNN backbone. The symbols “×” and “▴” represent the features extracted from source and target samples, respectively.

From the perspective of CNN backbones, ResNet34 performs best and VGG16 falls behind it slightly, followed by EfficientNet_B0. Here it is worth mentioning that EfficientNet_B0 shows the worst performance, which may be limited by its shrinking mechanism when there is not enough labeled data for training. Different from natural images containing a few focused objects, RS scene images involve numerous objects on the ground. Therefore, the shrinking architecture may lead to the discard of some useful information of RS images during the knowledge transfer process. In addition, to evaluated these CNN backbones more comprehensively, we make the comparison of our BSCA from three perspectives, including parameters, computing complexity (evaluated by multiply-accumulates, MACs), and mean classification accuracy, as shown in Table 8. The table reveals that EfficientNet_B0 has the minimal parameters and computational complexity, resulting the relatively low performance. On the contrast, benefiting from the efficient residual connection and maximum model parameters, ResNet34 achieves the best performance with the intermediate computing complexity.

From the side of types of methods, the SSL method of FixMatch performs worst with a dramatic gap than UDA and SSDA, verifying that domain adaptation strategy is more valuable for target domain classification of RS scene images than the semi-supervised learning strategy. For the UDA method of ECB-FAM, its performance is affected by source domains. For example, for the same target domain of NWPU-RESISC45 on VGG16, its accuracy decreases from 90.5% (AID is the source domain) to 74.6% (RSSCN7 is the source domain), with a gap of 15.9%. In contrast, as the combination of SSL and UDA, most of SSDA methods in experiments have more superior and stable performance, less influenced by source domains. The possible reason is that SSDA combines the advantages of UDA and SSL and therefore becomes more robust.

**Table 8:** Comparison of BSCA in parameters, computing complexity, and 12-adaptation-scenario mean accuracy based on different CNN models.

Model	Params (M) ↓	MACs (G) ↓	Mean accuracy (%) ↑
VGG16	14.98	15.35	93.0
ResNet34	21.55	3.68	**93.3**
EfficientNet_B0	**4.67**	**0.41**	91.8

From the point of view of SSDA methods, our BSCA achieves the best and most stable performance of all the SSDA methods tested. Compared with the basic method of S+T, SSDAN shows the meaningful improvement over ResNet34 and VGG16. However, SSDAN only obtains a small gain in EfficientNet_B0, which reveals its instability when facing different CNN backbones. ADDA has a poor performance, probably for its unique separated source and target extractors, which reveals the significance of model sharing across domains. RevGrad shows no benefit for performance, and ENT is even harmful for cross-domain classification in general. CDAC performs badly in RS-SSDA probably because the high overlapping objects and features across RS scene classes make the unlabeled feature-similarity-based clustering strategy of CDAC invalid; this phenomenon can be intuitively observed in its feature distribution provided in Section 4.2.

From the perspective of specific adaptation scenarios, the results from these experiments demonstrate that our BSCA can achieve the best knowledge transfer on most of the scenarios, except some scenarios where WHU serves as the target domain. The reason may be that its small number of samples limits the effectiveness of cross-domain alignment as well as its further classification performance.

In general, the experimental results demonstrate the effectiveness, superiority, and stability of the proposed BSCA when facing various RS adaptation scenarios and CNN backbones in comparison with SSL, UDA, and other state-of-the-art SSDA methods.

### 4.5 Visualization of feature distribution

To intuitively show the feature alignment effect of the proposed BSCA, in Fig. 7 the t-SNE technique (Van der Maaten and Hinton, 2008) is used to illustrate the feature distributions of our BSCA and other comparison methods in the adaptation scenario of N→A based on ResNet34.

The SSL method of FixMatch roughly aggregates the target features, but it ignores the utilization of the source features, resulting in the obvious cross-domain semantic gap. The UDA method of ECB-FAM decreases the cross-domain discrepancy, while the class boundary is not clear for some classes like “Residential” and “Industrial”. For SSDA methods, compared with the baseline of S+T, RevGrad shows little effect on reducing the domain shift, and the entropy minimization operation in ENT plays a negative role of making the cross-domain intra-class feature separated, which are consistent with its classification results. SSDAN works well by keeping the classes away from each other without interference to the cross-domain intra-class distribution. CDAC performs badly in RS-SSDA, probably influenced by its adaptive clustering method of unlabeled target feature based on the similarity of objects and feature covered in two images. However, there are plenty of object and feature overlapped among different RS scene classes, such as buildings and roads. As shown in Fig. 7(h), it can be seen that the feature of “‘Industrial” and “Residential” are largely mixed with each other, and therefore the unsupervised feature clustering makes their feature distribution unreasonably closer. In contrast, Fig. 7(i) reveals that our BSCA can not only increase the inter-class discrimination but also decrease the cross-domain intra-class distance, i.e., intra-class variance, and therefore achieve the best knowledge transfer performance, despite there being a few incorrect pseudo-labeled target samples. Overall, the visualizations demonstrate that our BSCA can achieve knowledge transfer from a source domain to a target domain via the cross-domain feature alignment.

## 5 Conclusion

In this paper, aiming at semi-supervised domain adaptation (SSDA) for remote sensing (RS) cross-domain scene classification, we design a bidirectional sample-class alignment (BSCA) method to reduce the semantic discrepancy between a source domain and a target domain. BSCA consists of two kinds of alignment mechanisms: unsupervised alignment (UA) and supervised alignment (SA). Firstly, the UA module concentrates on the inter-domain feature alignment in an unsupervised manner, not only between the source domain and the unlabeled target domain but also between the labeled target domain and the unlabeled target domain, which are achieved by decreasing their respective MMD distance. Then, the SA module aims to further reduce the domain shifts at the class level by the following sub-steps: calculate class-wise centers of two domains from the memory banks; make the sample-class feature alignment, both from source samples to their associated target class centers and from target samples from their associated source class centers; store the feature extracted from samples to the class-wise memory banks of two domains. In comparison with some state-of-the-art SSL/UDA/SSDA methods, our BSCA achieves superior classification performance on the unlabeled target domain with compact cross-domain intra-class feature representation and a low-entropy classification boundary. Such results demonstrate the effectiveness and robustness of the proposed method.

In future work, considering the performance bottleneck resulting from incorrect pseudo-labeled target samples in our BSCA, we will focus on improving the quality of pseudo-labels via dynamic threshold techniques such as curriculum learning.

## Declaration of Competing Interest

The authors declare that they have no known competing financial interests or personal relationships that could have appeared to influence the work reported in this paper.

## Footnotes

1 https://pytorch.org/.

2 https://github.com/VisionLearningGroup/SSDA_MME.

3 https://github.com/corenel/pytorch-adda.

4 https://github.com/fungtion/DANN.

5 https://github.com/lijichang/CVPR2021-SSDA.

## References

[b1] Ahmed, N., Saha, S., Shahzad, M., Fraz, M.M., Zhu, X.X., 2021. Progressive unsupervised deep transfer learning for forest mapping in satellite image. In: Proceedings of the IEEE/CVF International Conference on Computer Vision. pp. 752–761.

[b2] Alonso, I., Sabater, A., Ferstl, D., Montesano, L., Murillo, A.C., 2021. Semi-supervised semantic segmentation with pixel-level contrastive learning from a class-wise memory bank. In: Proceedings of the IEEE/CVF International Conference on Computer Vision. pp. 8219–8228.

[b3] Bai L., Liu Q., Li C., Ye Z., Hui M., Jia X. (2022). Remote sensing image scene classification using multi-scale feature fusion covariance network with octave convolution. IEEE Trans. Geosci. Remote Sens..

[b4] Borgwardt K.M., Gretton A., Rasch M.J., Kriegel H.P., Schölkopf B., Smola A.J. (2006). Integrating structured biological data by kernel maximum mean discrepancy. Bioinformatics.

[b5] Chen W.Y., Liu Y.C., Kira Z., Wang Y.C.F., Huang J.B. (2019). arxiv:1904.04232.

[b6] Cheng G., Han J., Lu X. (2017). Remote sensing image scene classification: Benchmark and state of the art. Proc. IEEE.

[b7] Cubuk, E.D., Zoph, B., Shlens, J., Le, Q.V., 2020. Randaugment: Practical automated data augmentation with a reduced search space. In: Proceedings of the IEEE/CVF Conference on Computer Vision and Pattern Recognition Workshops. pp. 702–703.

[b8] Deng J., Dong W., Socher R., Li L.J., Li K., Fei-Fei L. (2009). 2009 IEEE Conference on Computer Vision and Pattern Recognition.

[b9] Ganin Y., Ustinova E., Ajakan H., Germain P., Larochelle H., Laviolette F., Marchand M., Lempitsky V. (2016). Domain-adversarial training of neural networks. J. Mach. Learn. Res..

[b10] Grandvalet Y., Bengio Y. (2005). Semi-supervised learning by entropy minimization. CAP.

[b11] Gretton A., Borgwardt K.M., Rasch M.J., Schölkopf B., Smola A. (2012). A kernel two-sample test. J. Mach. Learn. Res..

[b12] Gretton A., Sejdinovic D., Strathmann H., Balakrishnan S., Pontil M., Fukumizu K., Sriperumbudur B.K. (2012). Advances in Neural Information Processing Systems.

[b13] Han W., Feng R., Wang L., Cheng Y. (2018). A semi-supervised generative framework with deep learning features for high-resolution remote sensing image scene classification. ISPRS J. Photogramm. Remote Sens..

[b14] He, K., Fan, H., Wu, Y., Xie, S., Girshick, R., 2020. Momentum contrast for unsupervised visual representation learning. In: Proceedings of the IEEE/CVF Conference on Computer Vision and Pattern Recognition. pp. 9729–9738.

[b15] He, K., Zhang, X., Ren, S., Sun, J., 2016. Deep residual learning for image recognition. In: Proceedings of the IEEE Conference on Computer Vision and Pattern Recognition. pp. 770–778.

[b16] Jiang P., Wu A., Han Y., Shao Y., Qi M., Li B. (2020). IJCAI.

[b17] Kim T., Kim C. (2020). European Conference on Computer Vision.

[b18] Lasloum T., Alhichri H., Bazi Y., Alajlan N. (2021). Ssdan: Multi-source semi-supervised domain adaptation network for remote sensing scene classification. Remote Sens..

[b19] Li D., Hospedales T. (2020). European Conference on Computer Vision.

[b20] Li, J., Li, G., Shi, Y., Yu, Y., 2021. Cross-domain adaptive clustering for semi-supervised domain adaptation. In: Proceedings of the IEEE/CVF Conference on Computer Vision and Pattern Recognition. pp. 2505–2514.

[b21] Lin M., Chen Q., Yan S. (2013). Network in network. Comput. Sci..

[b22] Long M., Cao Y., Wang J., Jordan M. (2015). International Conference on Machine Learning.

[b23] Lu X., Gong T., Zheng X. (2019). Multisource compensation network for remote sensing cross-domain scene classification. IEEE Trans. Geosci. Remote Sens..

[b24] Lu X., Gong T., Zheng X. (2020). Multisource compensation network for remote sensing cross-domain scene classification. IEEE Trans. Geosci. Remote Sens..

[b25] Lv, H., Chen, C., Cui, Z., Xu, C., Li, Y., Yang, J., 2021. Learning normal dynamics in videos with meta prototype network. In: Proceedings of the IEEE/CVF Conference on Computer Vision and Pattern Recognition. pp. 15425–15434.

[b26] Ma L., Li M., Ma X., Cheng L., Du P., Liu Y. (2017). A review of supervised object-based land-cover image classification. ISPRS J. Photogramm. Remote Sens..

[b27] Ma C., Sha D., Mu X. (2021). Unsupervised adversarial domain adaptation with error-correcting boundaries and feature adaption metric for remote-sensing scene classification. Remote Sens..

[b28] Ma A., Wan Y., Zhong Y., Wang J., Zhang L. (2021). Scenenet: Remote sensing scene classification deep learning network using multi-objective neural evolution architecture search. ISPRS J. Photogramm. Remote Sens..

[b29] Miao W., Geng J., Jiang W. (2022). Semi-supervised remote sensing image scene classification using representation consistency siamese network. IEEE Trans. Geosci. Remote Sens..

[b30] Motiian, S., Piccirilli, M., Adjeroh, D.A., Doretto, G., 2017. Unified deep supervised domain adaptation and generalization. In: Proceedings of the IEEE International Conference on Computer Vision. pp. 5715–5725.

[b31] Pan S.J., Tsang I.W., Kwok J.T., Yang Q. (2010). Domain adaptation via transfer component analysis. IEEE Trans. Neural Netw..

[b32] Paszke A., Gross S., Chintala S., Chanan G., Yang E., DeVito Z., Lin Z., Desmaison A., Antiga L., Lerer A. (2017).

[b33] Qiu C., Mou L., Schmitt M., Zhu X.X. (2019). Local climate zone-based urban land cover classification from multi-seasonal sentinel-2 images with a recurrent residual network. ISPRS J. Photogramm. Remote Sens..

[b34] Ranjan R., Castillo C.D., Chellappa R. (2017).

[b35] Rottensteiner F., Sohn G., Jung J., Gerke M., Baillard C., Benitez S., Breitkopf U. (2012). The isprs benchmark on urban object classification and 3d building reconstruction. ISPRS Ann. Photogram. Remote Sens. Spat. Inf. Sci..

[b36] Saito, K., Kim, D., Sclaroff, S., Darrell, T., Saenko, K., 2019. Semi-supervised domain adaptation via minimax entropy. In: Proceedings of the IEEE/CVF International Conference on Computer Vision. pp. 8050–8058.

[b37] Simonyan K., Zisserman A. (2014). arxiv:1409.1556.

[b38] Sohn K., Berthelot D., Carlini N., Zhang Z., Zhang H., Raffel C.A., Cubuk E.D., Kurakin A., Li C.L. (2020). Fixmatch: Simplifying semi-supervised learning with consistency and confidence. Adv. Neural Inf. Process. Syst..

[b39] Song S., Yu H., Miao Z., Zhang Q., Lin Y., Wang S. (2019). Domain adaptation for convolutional neural networks-based remote sensing scene classification. IEEE Geosci. Remote Sens. Lett..

[b40] Tan M., Le Q. (2019). International Conference on Machine Learning.

[b41] Tzeng, E., Hoffman, J., Saenko, K., Darrell, T., 2017. Adversarial discriminative domain adaptation. In: Proceedings of the IEEE Conference on Computer Vision and Pattern Recognition. pp. 7167–7176.

[b42] Van der Maaten L., Hinton G. (2008). Visualizing data using t-sne. J. Mach. Learn. Res..

[b43] Wang R., Collins L.M., Bradbury K., Malof J.M. (2018). IGARSS 2018-2018 IEEE International Geoscience and Remote Sensing Symposium.

[b44] Wang Q., Huang W., Xiong Z., Li X. (2020). Looking closer at the scene: Multiscale representation learning for remote sensing image scene classification. IEEE Trans. Neural Netw. Learn. Syst..

[b45] Wurm M., Stark T., Zhu X.X., Weigand M., Taubenböck H. (2019). Semantic segmentation of slums in satellite images using transfer learning on fully convolutional neural networks. ISPRS J. Photogramm. Remote Sens..

[b46] Xiong Y., Xu K., Dou Y., Zhao Y., Gao Z. (2021). Wrmatch: Improving fixmatch with weighted nuclear-norm regularization for few-shot remote sensing scene classification. IEEE Trans. Geosci. Remote Sens..

[b47] Xiong Z., Zhang F., Wang Y., Shi Y., Zhu X.X. (2022). arxiv:2210.04936.

[b48] Yu T., Lin J., Mou L., Hua Y., Zhu X., Wang Z.J. (2022). Scida: Self-correction integrated domain adaptation from single-to multi-label aerial images. IEEE Trans. Geosci. Remote Sens..

[b49] Yuan, Y., Wang, D., Wang, Q., 2019. Memory-augmented temporal dynamic learning for action recognition. In: Proceedings of the AAAI Conference on Artificial Intelligence. pp. 9167–9175.

[b50] Zhang J., Liu J., Pan B., Shi Z. (2020). Domain adaptation based on correlation subspace dynamic distribution alignment for remote sensing image scene classification. IEEE Trans. Geosci. Remote Sens..

[b51] Zheng X., Gong T., Li X., Lu X. (2021). Generalized scene classification from small-scale datasets with multitask learning. IEEE Trans. Geosci. Remote Sens..

[b52] Zheng J., Wu W., Yuan S., Zhao Y., Li W., Zhang L., Dong R., Fu H. (2022). A two-stage adaptation network (tsan) for remote sensing scene classification in single-source-mixed-multiple-target domain adaptation (s${}^{2}$m${}^{2}$t da) scenarios. IEEE Trans. Geosci. Remote Sens..

[b53] Zheng Z., Zhong Y., Su Y. (2022). Domain adaptation via a task-specific classifier framework for remote sensing cross-scene classification. IEEE Trans. Geosci. Remote Sens..

[b54] Zhu S., Du B., Zhang L., Li X. (2021). Attention-based multiscale residual adaptation network for cross-scene classification. IEEE Trans. Geosci. Remote Sens..

[b55] Zou Q., Ni L., Zhang T., Wang Q. (2015). Deep learning based feature selection for remote sensing scene classification. IEEE Geosci. Remote Sens. Lett..

